# Efficacy and safety of endoscopic ultrasound‐guided gallbladder drainage without dilation by using a 0.035‐inch stiff guidewire

**DOI:** 10.1002/deo2.327

**Published:** 2024-01-06

**Authors:** Michihiro Ono, Yuki Ikeda, Ginji Ohmori, Yohei Arihara, Ryo Shibuya, Atsushi Uesugi, Shutaro Oiwa, Ryo Ito, Makoto Usami, Michiko Yamada, Tomoyuki Abe, Masahiro Maeda

**Affiliations:** ^1^ Department of Pancreatobiliary Medicine Steel Memorial Muroran Hospital Hokkaido Japan; ^2^ Department of Gastroenterology Oji General Hospital Hokkaido Japan; ^3^ Department of Medical Oncology Sapporo Medical University School of Medicine Hokkaido Japan; ^4^ Department of Gastroenterology Steel Memorial Muroran Hospital Hokkaido Japan

**Keywords:** acute cholecystitis, endoscopy, dilation, drainage, stents

## Abstract

Endoscopic ultrasound‐guided gallbladder drainage for patients with cholecystitis and high surgical risk is commonly performed by dilating the fistula before inserting the delivery sheath; however, this carries an increased risk of peritonitis. To overcome this problem, we developed a new technique that did not require dilation, using a 0.035‐inch stiff guidewire, and retrospectively evaluated the efficacy and safety of this technique. This retrospective case series report collected data on non‐surgical patients who underwent endoscopic ultrasound‐guided gallbladder drainage for various indications at Steel Memorial Muroran Hospital between November 2020 and October 2022. A total of 71 patients were included (mean age 83 ± 7.6 years; 33 women and 38 men). Breakthrough of the delivery sheath without dilation of the fistula was successful in 97.2% (*n* = 69) of patients. The success rate of stent placement was 98.6% (*n* = 70), as was the clinical success rate. Complications occurred in 2.8% (*n* = 2) of patients. Early and late adverse events occurred in 2.8% (*n* = 2) and 12.7% (*n* = 9) of patients, respectively. The mean procedure time was 24.8 ± 9.3 min. If a 0.035‐inch stiff guidewire is used, the dilation procedure can be omitted in the endoscopic ultrasound‐guided gallbladder drainage using self‐expandable metal stents.

## INTRODUCTION

For patients with cholecystitis who are at high surgical risk, non‐surgical gallbladder drainage, including endoscopic ultrasound‐guided gallbladder drainage (EUS‐GBD), is the preferred treatment modality. Most prospective studies^1^, ^2^, ^3^, ^4^ that have validated the effectiveness of EUS‐GBD involve lumen‐apposing metal stents (LAMSs). In current practice, EUS‐GBD with LAMS is most commonly performed using the “hot technique,”^5^ which combines a cautery needle and stent into a single instrument to simplify the procedure. The hot technique is described as follows: i) puncture the gallbladder while cauterizing and ii) implant the stent at the same time. The hot technique is simple and safe; however, cautery dilation can cause a possible acute and late “burn effect” on the vessels around the needle tract, resulting in unexpected bleeding.^6^ On the other hand, the “cold technique”^5^ with self‐expandable metal stents (SEMSs) is commonly used in regions where LAMSs are not available as EUS‐GBD stents. The cold technique is described as follows: i) puncture of the gallbladder with a needle for EUS‐guided fine needle aspiration (EUS‐FNA), ii) contrast injection and placement of a guidewire, iii) replacement of the needle with a dilator, iv) dilation of the fistula, v) replacement of the dilator with the delivery sheath, vi) introduction of the delivery sheath into the gallbladder, and vii) deployment of the SEMS. However, this technique is complex and its safety has not been fully verified. The SEMS implantation method involves a dilation procedure that carries a risk of bile or intestinal fluid leakage before the stent is placed. To avoid this complication, we developed a new cold technique without the need for dilation using a 0.035‐inch stiff guidewire. This method makes it possible to omit steps iii), iv), and v) of the cold technique, which is expected to prevent bile leakage and reduce costs. This study aimed to evaluate the efficacy and safety of omitting the dilation procedure in EUS‐GBD by using a 0.035‐inch stiff guidewire.

## PROCEDURE OR TECHNIQUE

### Patients

This retrospective study included consecutive adult patients (age ≥18 years) with acute cholecystitis (AC) who were designated as having a high surgical risk and underwent EUS‐GBD between November 1, 2020, and October 31, 2022, at the Steel Memorial Muroran Hospital. The Institutional Review Board approved this study (No. J221202, UMIN000049625). Before undertaking the procedure, all patients were informed of the risks and benefits of EUS‐GBD and percutaneous transhepatic gallbladder drainage (PTGBD) as alternative therapies, and each patient provided written informed consent. According to the Tokyo Guidelines 2018 (TG18), AC was diagnosed based on clinical symptoms, signs of systemic inflammation, and computed tomography imaging findings and classified according to severity into three grades.^7^ Risk of surgery was assessed using age‐adjusted Charlson Comorbidity Index (ACCI) score and American Society of Anesthesiologists physical status classification (ASA‐PS) score, and ACCI≥6 or ASA‐PS≥3 for Grade 1 or 2 AC and ACCI≥4 or ASA‐PS≥3 for Grade 3 AC were defined as high surgical risk^8^ and eligible for gallbladder drainage. This study excluded patients with chronic cholecystitis or recurrent cholecystitis after non‐surgical treatment such as cases of tube occlusion after endoscopic transpapillary gallbladder drainage, in which a hard gallbladder wall was expected. Acalculous cholecystitis, hemorrhagic cholecystitis, and gallbladder torsion were also excluded. All procedures were performed by either an expert pancreato‐biliary endoscopist (Michihiro Ono) with experience in over 200 interventional EUS procedures or, under his supervision, by four endoscopists (Yuki Ikeda, Ginji Ohmori, Shutaro Oiwa, and Ryo Ito) specializing in endoscopy.

### Data collection

Clinical and procedural data were collected from electronic medical records, including comorbidity, ACCI score, ASA‐PS score, endoscopic data (type of echoendoscope, length of stent, procedural findings, and procedure time), technical success, complications, early and late adverse events (AEs), clinical success, and clinical course.

### Definitions of outcomes

The primary endpoint was the technical success rate, defined as the breakthrough of the delivery sheath without dilation of the fistula after guidewire placement. The secondary endpoints were the success rate of SEMS placement, safety, and clinical success rate. Safety was determined by the occurrence of complications, including bile leak with peritonitis, perforation, and significant bleeding, which warranted interventions such as blood product transfusion, endoscopic therapy, and radiological or surgical procedures. Additional AEs were defined as early (within 96 h) and late (after 96 h) AEs, including recurrence of cholecystitis, defined by TG18 as cholecystitis after achieving complete clinical remission, and other AEs, including stent migration. AEs were graded according to the American Society for Gastrointestinal Endoscopy guidelines.^9^ Procedure time was measured from scope insertion to removal. Clinical success was defined as the relief of cholecystitis symptoms and the improvement of laboratory data.^1^

### Procedure

**FIGURE 1:**
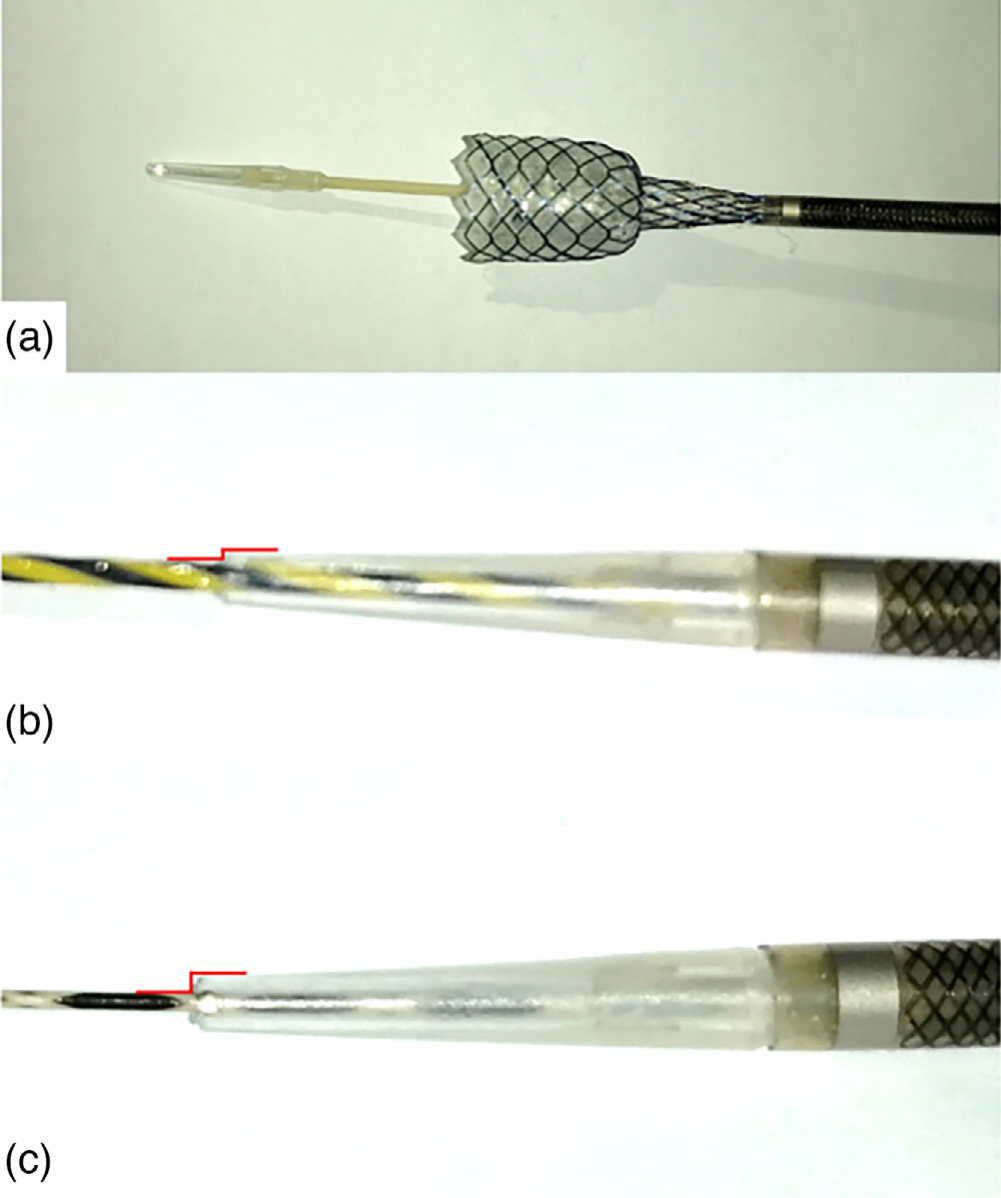
(a) BONASTENT M‐Intraductal (Medico's Hirata Inc.) is a 12‐mm fully covered self‐expandable metal stent (SEMS) with a central neckline 8 mm in diameter. (b) The step between the guidewire and the tip of the delivery sheath with a 0.035‐inch guidewire, there are fewer steps. (c) A 0.025‐inch guidewire would create a step and become trapped when breaking through the fistula.

Intravenous anesthesia and fluoroscopy were performed on all patients. The patients were administered intravenous antibiotic therapy and a computed tomography scan was performed immediately after the procedure or on the first postoperative day. EUS‐GBD was performed as follows. An echoendoscope (GF‐UCT260, Olympus or TGF‐UC260J; Olympus) connected to an ultrasound scanner (EU‐ME2; Olympus) was used to detect the gallbladder from the stomach or duodenum. Interposing vessels were identified using Doppler so that they could be avoided. A 19‐G needle for EUS‐FNA (EZ‐shot 3 plus; Olympus) was used to puncture the gallbladder through the working channel of the echoendoscope, and the aspirated bile was cultured. A contrast agent was injected to identify the gallbladder fluoroscopically, and a 0.035‐inch stiff guidewire (RevoWave‐α UltraHard; Piolax Medical Devices or RevoWave SeekMaster Hard; Piolax Medical Devices) was inserted. A 12 mm fully covered SEMS with a central neckline of 8 mm in diameter (Figure 1a, BONASTENT M‐Intraductal; Medico's Hirata Inc.) was introduced over the guidewire into the gallbladder without dilation of the fistula. The stent was deployed inside the working channel and implanted to bridge the gallbladder and gastrointestinal lumen.

### Follow‐up

SEMSs have largely been replaced with plastic stents, except in cases of malignancy or when the endoscopic procedure itself is invasive for the patient. SEMSs were removed if the gallstones were no longer observable on imaging. Outpatients who were able to visit the hospital continued to be followed up, and the others were contacted via telephone.

### Statistical analysis

All continuous variables were expressed as mean ± standard deviation and categorical variables were expressed as proportions (%). The sample size and statistical power were not calculated, as this was not a comparative study.

## RESULTS

**FIGURE 2:**
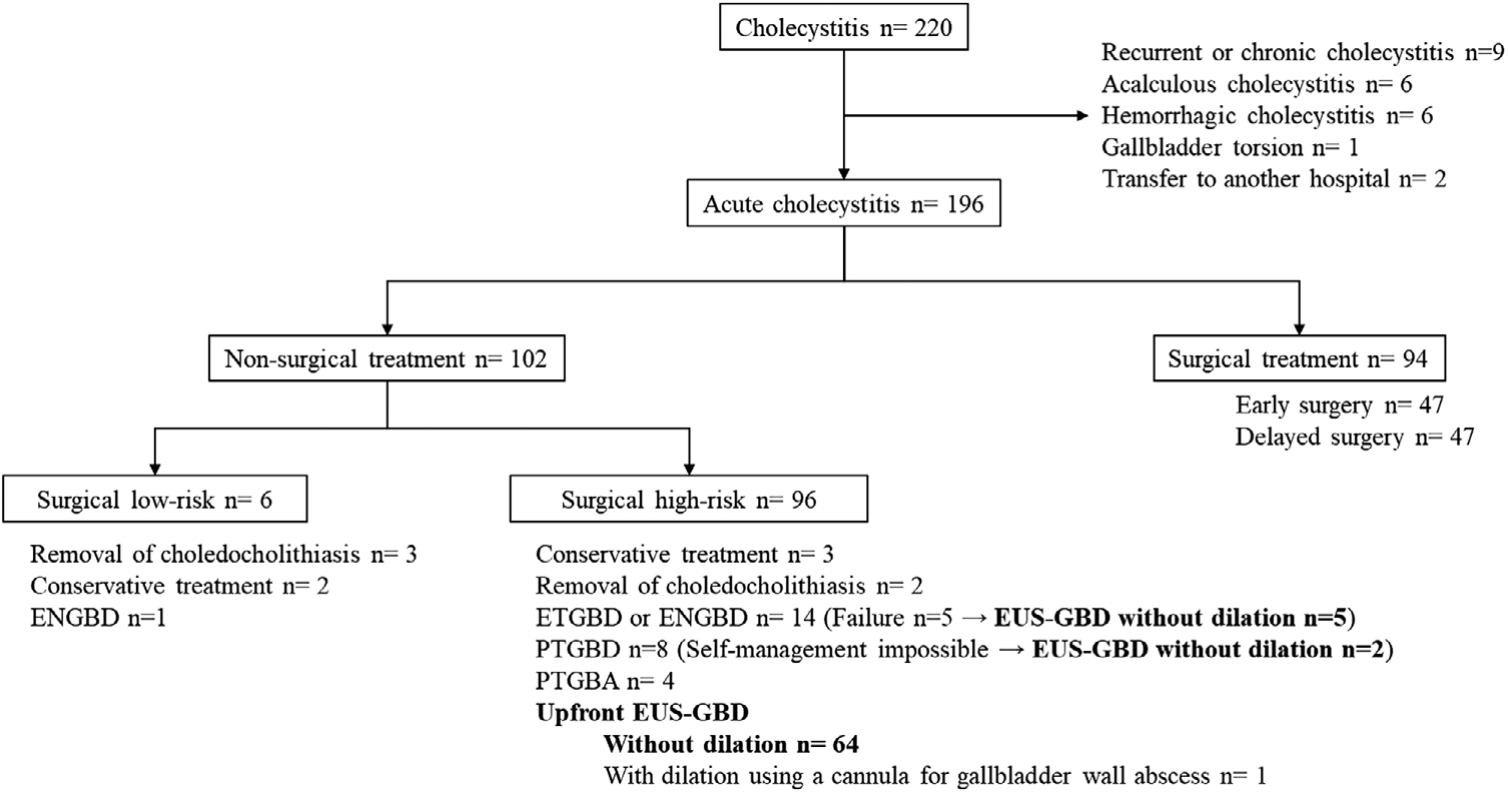
The flow chart, showing the treatment of patients with cholecystitis in this study. ENGBD, endoscopic naso‐gallbladder drainage; ETGBD, endoscopic transpapillary gallbladder drainage; EUS‐GBD, endoscopic ultrasound‐guided gallbladder drainage; PTGBA, percutaneous transhepatic gallbladder aspiration; PTGBD, percutaneous transhepatic gallbladder drainage.

During the study period, 220 patients with cholecystitis visited our hospital, 24 patients were excluded, 94 patients finally were resected gallbladder, while the remaining 102 patients were not. Out of the 96 patients deemed to be at high surgical risk, 65 patients underwent upfront EUS‐GBD, with one patient being excluded from this study due to the attempt to dilate using an ERCP cannula for gallbladder wall abscess. Five patients who failed ETGBD and two patients who were unable to self‐manage the PTGBD tube also underwent EUS‐GBD without dilation, resulting in a total of 71 patients included in this study (Figure 2). A total of 71 patients (33 women and 38 men) underwent EUS‐GBD. The patient characteristics are summarized in Table 1. The mean patient age was 83 ± 7.6 years, and the TG18 grades were G1 (*n* = 17), G2 (*n* = 43), and G3 (*n* = 11). The indication for EUS‐GBD was benign in 60 cases (*n* = 60) and malignant in 11 cases (*n* = 11). Mean ACCI was 8.1 ± 2.1, and ASA‐PSs were 1 (*n* = 12), 2 (*n* = 11), 3 (*n* = 35), and 4 (*n* = 13). Antithrombotic therapy (ATT) was administered to 54.9% (*n* = 39) of patients, single agents to 30 patients, multiple agents to nine patients, and ATT was discontinued in 35.9% (*n* = 14) prior to the procedure. Only one patient had undergone Billroth 2 gastrointestinal reconstructive surgery. This patient and one other patient had undergone PTGBD as prior treatment.

**TABLE 1:** Patient demographics.

			*N* = 71 (%)
Age, median ± SD			83 ± 7.6
Gender		Female	33 (46.5)
		Male	38 (53.5)
TG18 grade		G1	17 (23.9)
		G2	43 (60.6)
		G3	11 (15.5)
Cause of cholecystitis	Benign disease (*n* = 60)	Cholelithiasis	60 (84.5)
	Malignancy (*n* = 11)	Post biliary SEMS	6 (8.5)
		Biliary cancer	2 (2.8)
		Pancreatic cancer	2 (2.8)
		Post duodenal SEMS	1 (1.4)
ACCI, mean ± SD			8.1 ± 2.1
ASA‐PS		1	12 (16.9)
		2	11 (15.5)
		3	35 (49.3)
		4	13 (18.3)
Antithrombotic therapy			39 (54.9)
		SAPT	22
		Warfarin	3
		DOAC	5
		DAPT	4
		SAPT and DOAC	5
Gastrointestinal tract reconstruction methods		Billroth 2	1 (1.4%)
Prior PTGBD			2 (2.8%)

Abbreviations: ACCI, Age‐adjusted Charlson comorbidity index; DAPT, dual antiplatelet therapy; DOAC, direct oral anticoagulants; ASA‐PS, American Society of Anesthesiologists physical status classification; PTGBD, percutaneous transhepatic gallbladder drainage; SAPT, single antiplatelet therapy; SD, standard deviation; SEMS, self‐expandable metal; TG18, Tokyo Guidelines 2018.

The technical success rate was 97.2% (*n* = 69; Table 2), as in two cases the delivery sheath failed to break through and dilation was required. One case utilized a 6‐Fr cautery dilator (Cysto‐Gastro‐Sets; Endo‐Flex), while the other employed a 7‐Fr mechanical dilator (ES dilator; Zeon Medical Co., Ltd.). The success rate of SEMS placement was 98.6% (*n* = 70). The clinical success rate was 98.6% (*n* = 70).

**TABLE 2:** Procedure outcomes.

Technical success, *n* (%)		69 (97.2)
Procedure time, mean ± SD, min		24.8 ± 9.3
Echoendoscope	GF‐UCT260	52 (73.2)
	TGF‐UC260J	19 (26.7)
Initial puncture site	Duodenum	62 (87.3)
	Stomach	9 (12.7)
A portion of the gallbladder used	Neck	42 (59.2)
	Body	29 (40.8)
Stent length	40 mm	64 (90.1)
	50 mm	7 (9.9)
Clinical outcome	The success of placing SEMS, *n* (%)	70 (98.6)
	Clinical success, *n* (%)	70 (98.6)
Procedural‐related AE, severity grade		2 (2.8)
	Stent misdeploy requiring surgery, severe	1 (1.4)
	Pneumoperitoneum, mild	1 (1.4)
Early AE (within 96 h), severity grade		3 (4.2)
	Heart failure and pneumonia, mild	1
	Hemorrhagic cholecystitis, mild	1
Late AE (≥96 h)		9 (12.7)
	Stent migration	4
	Cholangitis	3
	Food impaction	1
	Bleeding	1

Abbreviations: AE, adverse event; SD, standard deviation; SEMS, self‐expandable metal stent.

Complications occurred in 2.8% (*n* = 2) of the patients. In one patient, we were able to break through the delivery sheath without dilation, but the stent failed to deploy, requiring emergency surgery. The other patient developed pneumoperitoneum immediately after stent deployment; however, there was no abdominal pain, and peritonitis did not develop. The procedure‐related mortality was nil. Early AEs occurred in 2.8% (*n* = 2) of patients. One patient had simultaneous heart failure and pneumonia, and the other developed hemorrhagic cholecystitis due to a Vitamin K deficiency caused by long‐term antibiotic therapy; however, both improved with conservative treatment. Late AEs occurred in 12.7% (*n* = 9) of patients. Of these, four (*n* = 4) had a migrated stent, two of which improved without additional stenting, and two improved after reinsertion of a plastic stent. Three (*n* = 3) developed cholangitis caused by obstruction from stents implanted for bile duct stones (*n* = 1) and bile duct cancer (*n* = 2), both of which were treated endoscopically and improved. One (*n* = 1) developed food impaction, the SEMS was removed, and the contents of the gallbladder and stones were completely removed with endoscopy. One (*n* = 1) developed bleeding that occurred 3 months later, which was ceased endoscopically.

The mean procedure time was 24.8 ± 9.3 min. The echoendoscope types were GF‐UCT260 (*n* = 52) and TGF‐UC260J (*n* = 19), and in one case for each endoscope, the delivery sheath did not break through without dilation. The initial puncture sites were the duodenum (*n* = 62) and stomach (*n* = 9), and in the two cases that required dilation for the breakthrough of the delivery sheath, both puncture sites were in the duodenum. The portion of the gallbladder used for drainage was the neck (*n* = 42) and body (*n* = 29), with one case each requiring dilation.

SEMSs were removed in 31.0% (*n* = 22). Of these, in three patients, stents were not reinserted, and in 19 they were replaced with plastic stents. Stent removal and replacement were performed based on each patient's general condition and wishes. Cholecystitis recurred in 7% (*n* = 5) of the patients during the follow‐up period. The causes of death are summarized in Table 3. Mortality was 15.5% (*n* = 11), and the etiologies included cancer (*n* = 4), chronic renal failure (*n* = 2), heart failure (*n* = 1), respiratory failure (*n* = 1), liver failure (*n* = 1), ventricular fibrillation (*n* = 1), and pneumonia (*n* = 1); no deaths were attributed to cholecystitis. The mean time from EUS‐GBD to death was 82.9 ± 62.8 days.

**TABLE 3:** Clinical course.

	*N* = 71 (%)
Removal of SEMS	22 (31.0)
No stent	3
Convert to plastic stent	19
Recurrent of cholecystitis	5 (7.0)
Mortality	11 (15.5)
Cause of death	
Cancer	4
Chronic renal failure	2
Heart failure	1
Respiratory failure	1
Liver failure	1
Ventricular fibrillation	1
Pneumonia	1
Time from EUS‐GBD to death, mean ± SD, days	82.9 ± 62.8

Abbreviations: EUS‐GBD, endoscopic ultrasound‐guided gallbladder drainage; SD, standard deviation; SEMS, self‐expandable metal stent.

## DISCUSSION

According to the TG18, laparoscopic cholecystectomy is recommended for patients with low CCI or ASA‐PS, indicating a low surgical risk profile.^8^ The CCI was first proposed by Charlson et al. in 1987 to account for the influence of patients’ complications and is useful for prognostic prediction by weighting and scoring each comorbid disease.^10^ Furthermore, Charlson et al. proposed an ACCI, showing that the estimated relative risk of death from an increase of one in the comorbidity score is equal to that from an additional decade of age.^11^ In this study, ACCI≥6 or ASA‐PS≥3 for Grade 1 or 2 AC and ACCI≥4 or ASA‐PS≥3 for Grade 3 AC were defined as surgical‐high risk and eligible for gallbladder drainage.

Most prospective EUS‐GBD reports^1^, ^2^, ^3^, ^4^ are available for LAMSs. More recently, a hot technique^5^ using a stent delivery system with a cautery tip, first reported in 2014,^12^ has been widely utilized. Despite its effectiveness, previous studies have reported rare but severe intraprocedural AEs.^13^, ^14^, ^15^ In contrast, the cold technique^5^ without cautery dilation using SEMSs is performed in countries where LAMSs are not available for EUS‐GBD. However, the cold technique generally involves mechanical dilation of the fistula before insertion of the SEMS, which raises concerns regarding bile leakage and peritonitis. Ogura et al.^16^ comprehensively reviewed EUS‐GBD, summarizing outcomes with LAMS and SEMS or plastic stents. They reported a single case of bile peritonitis with LAMS, while, despite fewer reported cases with SEMS or plastic stents, there were nine pneumoperitoneum cases and one bile peritonitis case. Considering the primary distinction between these techniques is the presence or absence of dilation, we suggest that the dilation step may contribute to the heightened risk of bile peritonitis. Computed tomography scans were used for precise assessment of bile leakage the following day, revealing only one case of pneumoperitoneum without peritonitis. The use of a stiff guidewire reduces the need for fistula dilation, preventing bile leakage in the cold technique. Additionally, this method is cost‐effective, eliminating the risk of a late burn effect associated with the hot technique.

The safety of EUS‐GBD in patients receiving ATT has been previously reported.^17^ In our study, half of the patients were on ATT, and even though two patients had hemorrhage, none of them were determined to be directly related to ATT. One case was spurting bleeding due to a mechanically induced ulcer of the gallbladder wall by the SEMS, which was cauterized using hemostatic forceps.^18^

The occurrence of bile leakage in EUS‐GBD is more likely as compared with EUS‐HGS as a result of the lack of tamponade effect by the liver.^16^ Consequently, we chose a fully covered SEMS rather than a plastic stent. We used a braided SEMS with an 8Fr delivery sheath and a central neckline (Figure 1a, BONASTENT M‐Intraductal). It allows for the endoscopic ultrasound image to confirm the central neckline during implantation, which can prevent intra‐gallbladder and intra‐abdominal deployment. Additionally, the central neckline is expected to prevent the late deviation of the SEMS; however, four cases of stent migration were identified, so there is still room for improvement.

One of the factors that may contribute to the difficulty of breaking through the delivery sheath is the decrease in the transmitting force. The forward‐viewing echoendoscope TGF‐UC260J may solve this problem; however, the delivery sheath could not be broken through in one of the 19 cases in which it was used. Elderly patients have a higher frequency of Chilaiditi's syndrome or steerhorn stomach, which causes gastrointestinal deformities that may make it difficult to transmit force. If the guidewire cannot be sufficiently delivered into the gallbladder and the scope is not well positioned, it may be better to dilate the fistula.

According to previous reports, there is no evidence of significant clinical differences between the duodenum and stomach puncture sites.^19^, ^20^ Despite concerns about transgastric puncture without dilation, it was successfully performed in all cases. The thicker stomach mucosa and challenges in force transmission due to the unfixed gallbladder did not necessitate dilation, contrary to expectations. Ogura et al.^16^ suggested that the duodenum may have less mobility than the stomach, potentially resulting in a lower risk of stent migration with the transduodenal approach. However, in our study, three out of four cases of migration occurred with the transduodenal approach. Therefore, further research is necessary to comprehensively evaluate the safety profile of each puncture site.

Technical tips for this procedure include patient selection, the scope position, the use of a 0.035‐inch stiff guidewire, attention to the direction of placement of the guidewire, and careful consideration of the puncture site. The elevated procedural success rates and low incidence of complications in our study are primarily attributed to meticulous patient selection, particularly the exclusion of cases with chronic or recurrent cholecystitis. The use of a 0.035‐inch guidewire eliminates the step between the guidewire and the tip of the delivery sheath (Figure 1b, c), which facilitates the breakthrough of the gastrointestinal and gallbladder mucosa. However, it's important to note that when we refer to a ‘0.035‐inch guidewire’, we specifically mean a guidewire thinner than 0.035 inches. It should be emphasized that some other 0.035‐inch guidewires may not successfully pass through the 19‐gauge FNA needle. There is sometimes strong resistance when pushing the 0.035‐inch guidewire into the FNA needle, and it may not be possible to coil it within the gallbladder. In such situations, only the FNA needle is carefully removed, and the stiffness of the guidewire allows it to be pushed in and coiled (Supporting Information). In this context, optimal guidewire placement is crucial, ensuring smooth force application. Puncture site selection and pre‐puncture EUS findings are critical, emphasizing avoidance of the gallbladder fundus due to its tendency to move away from the duodenum when collapses. The gallbladder neck is a common puncture site; however, caution is warranted due to potential limitations in space for deploying SEMS.

The limitations of the current study include its single‐center retrospective design, and the lack of a comparison group such as EUS‐GBD using a conventional 0.025‐inch guidewire. All EUS‐GBD procedures in this study were performed under the supervision of specialists; therefore, this procedure should be performed with great caution by inexperienced endoscopists.

In conclusion, the dilation procedure can be omitted by using a 0.035‐inch stiff guidewire in EUS‐GBD with the cold technique, thus possibly preventing bile leakage into the abdominal cavity and reducing costs .

## CONFLICT OF INTEREST STATEMENT

The authors declare no conflict of interest.

## Supporting information



A video shows the recommended technique, addressing the nuances of the procedure, including the careful removal of the FNA needle, seeking and coiling the guidewire, and placing the SEMS.
